# Use of FGF-21 as a Biomarker of Mitochondrial Disease in Clinical Practice

**DOI:** 10.3390/jcm6080080

**Published:** 2017-08-21

**Authors:** Alireza Morovat, Gayani Weerasinghe, Victoria Nesbitt, Monika Hofer, Thomas Agnew, Geralrine Quaghebeur, Kate Sergeant, Carl Fratter, Nishan Guha, Mehdi Mirzazadeh, Joanna Poulton

**Affiliations:** 1 Department of Clinical Biochemistry, Oxford University Hospitals, Oxford OX3 9DU, UK; reza.morovat@ouh.nhs.uk (A.M.); gayani.weerasinghe@ouh.nhs.uk (G.W.); nishan.guha@ouh.nhs.uk (N.G.); mehdi.mirzazadeh@nhs.net (M.M.); 2 Department of Paediatrics, The Children’s Hospital, Oxford OX3 9DU, UK; victoria.nesbitt@ouh.nhs.uk; 3 Department of Neuropathology and Ocular Pathology, West Wing, Oxford University Hospitals, Oxford OX3 9DU, UK; monika.hofer@ouh.nhs.uk; 4 Sir William Dunn School of Pathology, University of Oxford, Oxford OX1 3RE, UK; thomas.agnew@path.ox.ac.uk; 5 Department of Neuroradiology, West Wing, Oxford University Hospitals, Oxford OX3 9DU, UK; gerardine.quaghebeur@ouh.nhs.uk; 6 NHS Specialised Services for Rare Mitochondrial Disorders of Adults and Children UK, Oxford Medical Genetics Laboratories, Oxford University Hospitals, Oxford OX3 7LE, UK; kate.sergeant@ouh.nhs.uk (K.S.); carl.fratter@ouh.nhs.uk (C.F.); 7 Nuffield Department of Obstetrics and Gynaecology, University of Oxford, Oxford OX3 9DU, UK

**Keywords:** fibroblast growth factor-21, FGF-21, mitochondrial disease, diagnosis

## Abstract

Recent work has suggested that fibroblast growth factor-21 (FGF-21) is a useful biomarker of mitochondrial disease (MD). We routinely measured FGF-21 levels on patients who were investigated at our centre for MD and evaluated its diagnostic performance based on detailed genetic and other laboratory findings. Patients’ FGF-21 results were assessed by the use of age-adjusted *z*-scores based on normalised FGF-21 values from a healthy population. One hundred and fifty five patients were investigated. One hundred and four of these patients had molecular evidence for MD, 27 were deemed to have disorders other than MD (non-MD), and 24 had possible MD. Patients with defects in mitochondrial DNA (mtDNA) maintenance (*n* = 32) and mtDNA rearrangements (*n* = 17) had the highest median FGF-21 among the MD group. Other MD patients harbouring mtDNA point mutations (*n* = 40) or mutations in other autosomal genes (*n* = 7) and those with partially characterised MD had lower FGF-21 levels. The area under the receiver operating characteristic curve for distinguishing MD from non-MD patients was 0.69. No correlation between FGF-21 and creatinine, creatine kinase, or cardio-skeletal myopathy score was found. FGF-21 was significantly associated with plasma lactate and ocular myopathy. Although FGF-21 was found to have a low sensitivity for detecting MD, at a *z*-score of 2.8, its specificity was above 90%. We suggest that a high serum concentration of FGF-21 would be clinically useful in MD, especially in adult patients with chronic progressive external ophthalmoplegia, and may enable bypassing muscle biopsy and directly opting for genetic analysis. Availability of its assay has thus modified our diagnostic pathway.

## 1. Introduction

Mitochondrial disease (MD) is common, with 1 in 400 individuals harbouring the m.3243A>G mutation that usually causes mild disease such as presbycusis [1]. The vast majority of these are never identified, hence the reported prevalence of around 1:20,000 in children and 1:8000 in adults [2,3]. Mitochondrial diseases are notoriously difficult to diagnose because of their heterogeneity, the presence of mitochondrial heteroplasmy, poor genotype-phenotype relationships, and the bluntness of the generally used laboratory tests for assessing mitochondrial dysfunction. The diagnosis of mitochondrial disease often involves an invasive muscle biopsy for studies of mitochondrial function. Respiratory chain enzyme studies are technically demanding and available only in a few specialist centres. Furthermore, they may generate false positive results if tissue samples are poorly preserved [4]. The diagnosis usually requires confirmatory genetic analysis. Conventional blood tests, such as plasma lactate, pyruvate, and creatine kinase (CK), are too insensitive and non-specific to be of significant help in most cases. Depending on the presentation, amino acids, acylcarnitines, and urine organic acids results may help modify the index of suspicion, but their value is very limited. 

Recent studies on serum fibroblast growth factor-21 (FGF-21) have suggested that it may offer a much better diagnostic power than conventional serum tests [5,6,7]. FGF-21 is a hormone-like cytokine that is involved in intermediary metabolism of carbohydrates and lipids [8]. Unlike the mouse, in which hepatic FGF-21 is induced by peroxisomal proliferator-activated receptor-α (PPARα) and in response to fasting [9,10], the function of FGF-21 in humans is still not completely understood. Circulating FGF-21 is mainly hepatic in origin in humans, but the protein is also expressed in adipocytes, myocytes, and the pancreas [11,12,13]. Some investigators hold that myocytic FGF-21 is induced as part of a protective mechanism against metabolic stress [14,15], although this is controversial [16]. Patients with respiratory chain deficiency have an increased myocytic expression of FGF-21, and treatment of myoblasts with FGF-21 increases PPARδ coactivator-1α (PGC-1α), thereby increasing ATP synthesis through the mammalian target of rapamycin—Yin Yang 1—PPAR gamma coactivator-1α (PGC-1α) pathway [17]. The induction of PGC-1α has lipolytic effects, and systemic FGF-21 improves glucose tolerance by stimulating insulin independent glucose-uptake in human myocytes and adipocytes [18,19]. FGF-21 was shown to increase in mitochondrial dysfunction and myopathy secondary to iron-sulphur cluster deficiency [20]. In trying to elucidate the mechanism linking FGF-21 and mitochondrial stress, other studies have found myocytic FGF-21 expression to be controlled by myogenic factor MyoD and to be driven by mitochondrial reactive oxygen species [21].

In the light of previous data showing that FGF-21 may be useful in a subgroup of patients with mitochondrial disease [5], in 2013 we adopted measurement of FGF-21 as an adjunct to first-line routine laboratory tests. This was aimed at improving diagnosis of patients in whom there was a high index of suspicion for the presence of mitochondrial disease, with a view to reducing the need for muscle biopsy in the assessment of patients. This report describes our findings.

## 2. Methods

In 2013, the Department of Clinical Biochemistry at the John Radcliffe Hospital in Oxford started offering FGF-21 assay to specialist clinicians for assessing patients who were being investigated for mitochondrial disease. The service was offered based on previous publications suggesting that FGF-21 is a useful marker for mitochondrial disease compared with other routine biochemical tests. We have retrospectively reviewed the FGF-21 data in the light of genetic findings and final diagnoses.

### 2.1. Individual Populations and Investigations

We reviewed all adult and paediatric patients referred to the Oxford centre for rare mitochondrial disorders at Oxford University Hospitals (OUH) between June 2013 and February 2017. Referrals were either from OUH consultants or from other counties in the United Kingdom. Patients were referred largely from clinical geneticists, neurologists, paediatricians, endocrinologists, and ophthalmologists because of suspected mitochondrial disease. In many cases, the DNA investigations were carried out prior to each individual being seen in the clinic. The referred patients were reviewed by a mitochondrial genetics consultant and her team and were then investigated further, usually while attending an outpatient appointment. Patients’ demographics were recorded, and clinical details were reviewed extensively with special attention to the presence of ocular myopathy (ptosis or progressive external ophthalmoplegia (PEO)), cardio-skeletal myopathy, hearing loss, neuropathy, enteropathy, epilepsy, brain-stem signs and symptoms, diabetes, and liver disease.

Following clinical assessments, investigations carried out were routine biochemical tests that included plasma CK, lactate, and FGF-21. A small number of patients had their FGF-21 repeated during follow-up. Depending on clinical presentation, many patients were investigated further with a muscle biopsy that frequently included respiratory chain enzyme studies. Furthermore, genetic testing was offered to all patients, often being undertaken before clinical review in the specialist clinic.

Patients with a confirmed genetic diagnosis of mitochondrial disease (MD) were grouped according to mitochondrial DNA point mutation, single DNA rearrangement, defects in mitochondrial maintenance, partly-characterised mitochondrial myopathy, and other autosomal MD. All (i) patients in whom alternative non-mitochondrial diagnoses (such as inclusion body myositis, optic neuritis and tyrosine hydroxylase deficiency) had been made, (ii) patients who lacked biochemical, molecular, and clinical features of mitochondrial disease and were hence discharged from the clinic, and (iii) unaffected relatives were all classified as non-mitochondrial disease (non-MD) group. Patients with no identifiable genetic mutation but with either (i) muscle biopsies suggestive of mitochondrial disease (respiratory chain function and/or muscle histochemistry) or (ii) where biochemical, molecular, and/or clinic features of mitochondrial disease were sufficient for inclusion as likely mitochondrial disease in the NHS England’s 100,000 genome study were classed as “possible-MD”.

Data from clinical case notes and electronic records, the laboratory information system, and mitochondrial genetic reports were collected by clinicians directly involved in the care of patients.

Ethics approval and consent were obtained for collecting specimens for FGF-21 measurement from a population of 28 children who had been diagnosed with congenital hypothyroidism but had been on successful treatment and were shown to be euthyroid, and separately from 50 healthy adult volunteers.

### 2.2. Laboratory Analyses

Routine biochemical tests were performed by the use of verified automated methods. Respiratory chain enzyme studies were performed by the University College London Neurometabolic Unit at the National Hospital for Neurology and Neurosurgery.

Genetic analyses were undertaken under the Highly Specialised NHS Service for Rare Mitochondrial Disorders of Adults and Children, largely by the Molecular Genetics Laboratory at the Churchill Hospital, Oxford, but some specimens were referred to the arms of the service operating in Newcastle or London. This included exome sequencing, which was carried out as a research project funded by Lily Foundation, based at Guy’s and St Thomas’ NHS Foundation Trust, London, UK. Histopathology on muscle biopsies was performed at the Neuropathology Department at the John Radcliffe Hospital.

Specimens for FGF-21 were centrifuged, and serum was separated and stored at −80 °C until analysis. FGF-21 was measured by ELISA (BioVendor, Brno, Czech Republic) according to the manufacturer’s instructions, with the exception of a reduced incubation time with the substrate (the final step) in order to prevent top calibrants’ absorbances reaching values above 2.5 units. We have unpublished data to indicate that FGF-21 is stable for up to three days in unseparated and separated serum, and its assay is unaffected by haemolysis. In our hands, the assay had an inter-batch coefficient of variation (CV) of ≤8.0% at concentrations of 108–2642 ng/L.

### 2.3. Statistics

The Biovendor’s FGF-21 kit insert describes FGF-21 mean and SD values from a healthy population. Since these data indicated a skewed distribution, we requested and were kindly supplied with the raw data, from which we were able to normalise them, derive SD, and assess patients’ FGF-21 values based on *z*-scores. Analyse-it software (Analyse-it Software Ltd, Leeds, UK) was used for all statistical analyses. Anderson-Darling A2 normality test was used to assess the healthy population’s descriptive data. Logarithmic transformation was found to be the best mode for normalising the data, and was also employed to assess the distribution of values separated into various age bins. Mean and SD of transformed data were calculated. To assess patients’ FGF-21 values, they were similarly log-transformed, and their *z*-scores calculated from the number of SDs above or below the mean for each age bin. In practice, values with *z*-scores of ≥2 were considered clinically significant. Receiver operating characteristic (ROC) curves were used to assess the performance of FGF-21 *z*-scores for diagnosing MD. Comparisons of values between different groups of patients, and also between kit manufacturer’ and Oxford’s healthy adult and paediatric populations were made by the Mann-Whitney test. Association between variables was assessed by linear regression.

## 3. Results

### 3.1. FGF-21 Reference Values

Serum FGF-21 raw data from a healthy population (76 men and 108 women; age range 2–85 years) were obtained from the manufacturer of FGF-21 kits. The data showed serum concentrations to range from undetectable to 1210 ng/L, with a distribution that had a skewness of 2.01 and a kurtosis of 5.48 (*p* < 0.0001). There was no difference between FGF-21 values in men and women (*p* = 0.907), but FGF-21 showed an increase with age (mean 3.0 ng/L per year (95% CI: 1.4–4.6 ng/L per year); *p* < 0.0001). Dividing values into age groups 21–60 and >60 years gave log-transformed distributions that were not significantly different from normal (*n* = 97, skewness −0.49, *p* = 0.056, and *n* = 68, skewness −0.07, *p* = 0.628, respectively). Log-transformed mean and SD values were 2.21 and 0.383 for the age group 21–60 years, and 2.41 and 0.296 for the age group >60 years. The number of healthy individuals aged ≤20 years was only 16, and the addition of these to the 21–60 years age group resulted in a significant negative skew in the log-transformed distribution.

FGF-21 values obtained on Oxford’s healthy adults and children were significantly lower than those supplied by the kit manufacturer (*p* < 0.0001 and *p* = 0.0013, respectively). Oxford’s healthy adults (age range 19–68 years) and children (age range 1–15 years) had log-transformed mean (SD) values of 1.93 (0.499) and 1.68 (0.422), respectively. In view of this difference and despite fewer numbers of individuals in the Oxford’s group, these data were used to establish FGF-21 *z*-scores for patients’ populations.

### 3.2. Patients’ Demographics and Diagnoses

One hundred and eighty four patients were investigated. Of these, 29 patients were omitted from our data analyses as insufficient clinical information related to diagnosis was available. The remaining 155 patients were aged between one day and 87 years at the time of investigations, with mean and median age of 38.0 and 37.5 years, respectively.

Diagnosis of mitochondrial disease was made in 104 patients (MD group) based on DNA data (Table 1). The category of patients with mitochondrial DNA (mtDNA) point mutations was the largest (40 cases), with m.3243A>G mutation comprising the majority (29 patients). The category of mtDNA maintenance defect had 32 cases, including 12 DNA polymerase subunit gamma (*POLG*), 6 ribonucleoside-diphosphate reductase subunit M2 B (*RRM2B*) and 5 *TWNK* gene defects. Single mtDNA rearrangements constituted 17 cases. There were 27 patients, who did not have mitochondrial defect (non-MD group). In nine of these 27, a clearly defined diagnosis was made, and this was mostly a metabolic enzyme defect. In a further 14 of the non-MD patients, a diagnosis of MD was deemed clinically unlikely, and there were a further four individuals in the non-MD group who were unaffected relatives of patients. Finally, there were 24 patients in whom a definitive diagnosis could not be made, but these patients were deemed likely to have MD, often on the basis of muscle biopsy histochemistry (possible-MD group). Patients in the possible-MD group were much younger than those in either the MD or the non-MD groups (median ages 11.0, 39.5, and 45.0 years, respectively; Table 1). 

### 3.3. Biochemical Data

**Figure 1:**
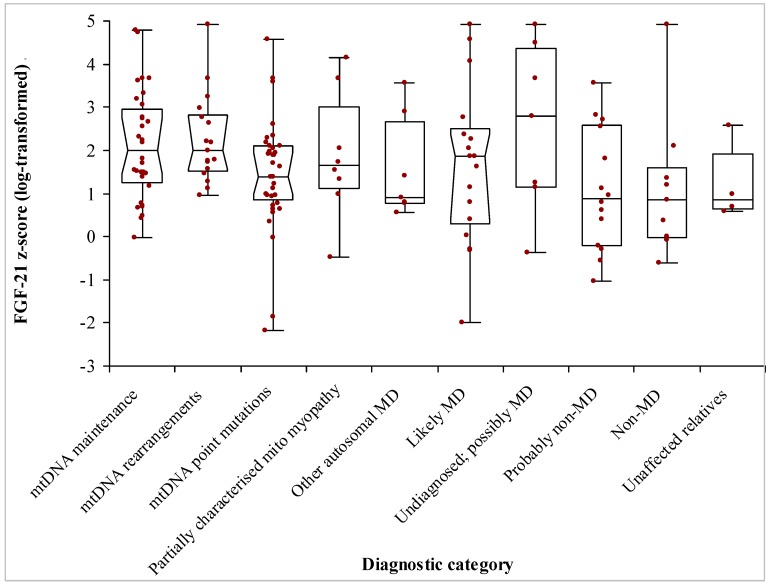
FGF-21 *z*-scores obtained on specimens from 155 patients investigated for mitochondrial disease. FGF-21 values were normalised by log-transformation, and *z*-scores were calculated based on age-dependent distributions. Patients were divided into a mitochondrial disease (MD) group consisting of those with mitochondrial DNA or maintenance defects, 30 patients without MD, and 24 patients who were deemed likely to have MD.

The laboratory reported serum FGF-21 concentrations up to a value 5700 ng/L and the exact concentrations above this upper limit were not quantified. Thus, the highest FGF-21 *z*-scores of 3.67 for adults and 4.91 for children, relate to FGF-21 values of 5700 ng/L. Patients with mitochondrial rearrangements or defects in mitochondrial maintenance had the highest FGF-21 (median *z*-score of 1.99 for both groups) (Figure 1 and Table 1). On the other hand, patients with defects in mitochondrial dynamics (mostly *OPA1* and *MFN2* gene defects) had the lowest FGF-21 *z*-scores (*n* = 7; median 0.90; “other autosomal MD” category in Figure 1). Many of the patients with m.3243A>G point mutation had FGF-21 values that were below those of non-MD patients. FGF-21 was neither predictable by any single clinical feature nor by the degree of heteroplasmy in blood.

**Figure 2:**
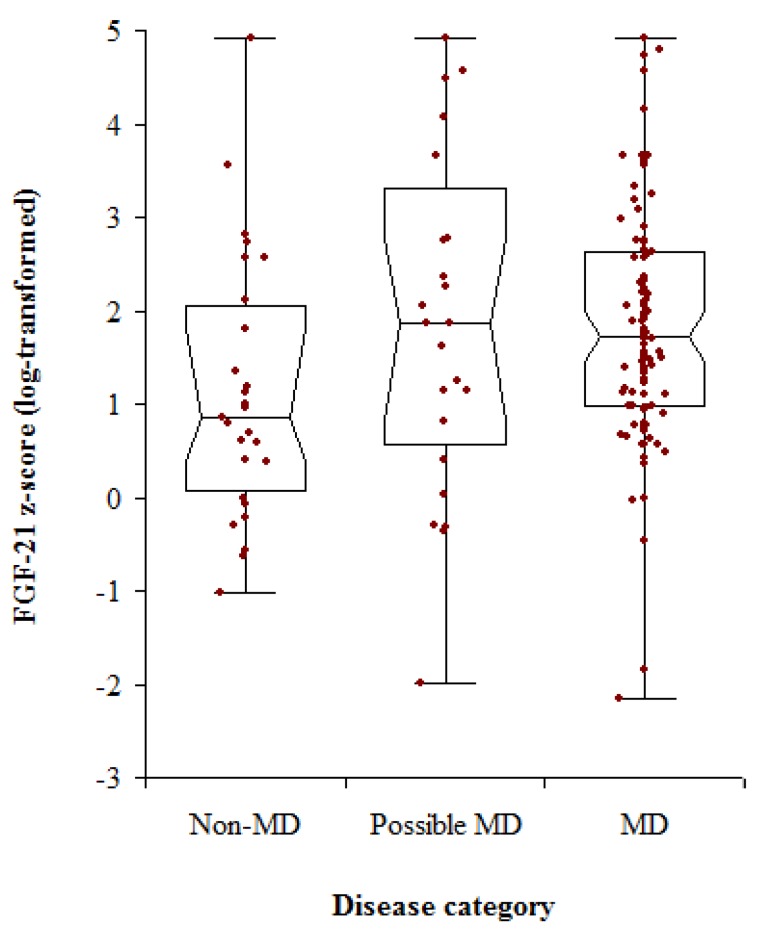
Distribution of FGF-21 *z*-scores in patients investigated for mitochondrial disease (MD). Patients were grouped according to the final diagnoses, with those who were likely to have MD but whose further investigations were still pending placed in the possible MD group.

**Figure 3:**
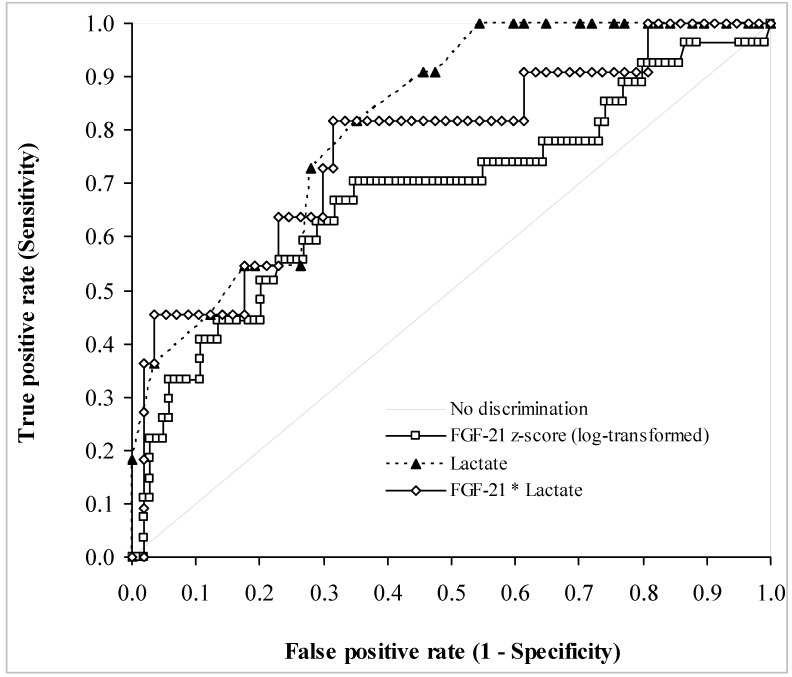
Receiver operating characteristic curves comparing FGF-21 *z*-scores, lactate concentration, and the product of FGF-21 and lactate concentrations for the diagnosis of mitochondrial disease. The curves were established using data from 104 MD and 27 non-MD patients. See Table 2 for further details on the diagnostic performance of FGF-21 *z*-scores.

As a group, patients with MD had a median FGF-21 *z*-score of 1.72, compared with 0.86 for non-MD patients (*p* = 0.0037). The median FGF-21 *z*-score for patients with possible MD was 1.87, comparable with the MD group (*p* = 0.852), but these patients also had a wide range of values (Table 1 and Figure 2). Using MD and non-MD patients’ FGF-21 *z*-scores for ROC analysis gave an area under the curve that was 0.68 (Figure 3). A *z*-score of 2.8 was associated with a specificity of 0.93 but a sensitivity of only 0.20 (Table 2). The assay had positive predictive values that were around 0.90, but poor negative predictive values (Table 2). Given the high median FGF-21 in patients with mtDNA maintenance and rearrangement defects, we assessed the power of the test for discriminating between these patients and the non-MD group.

There were 39 patients in whom myopathy was a prominent feature (assessed clinically as we do not routinely measure respiratory chain activities in adult patients), but there was no difference between the FGF-21 *z*-scores in these patients and in the ones without myopathy (*p* = 0.320; Table 1). Also, patients who had ptosis and/or PEO also had median FGF-21 *z*-score that were comparable with those in patients without ocular myopathy (*p* = 108; Table 1). Repeated measurements of FGF-21 in 10 out of 11 patients suggested that FGF-21 concentrations were fairly stable, with a good correlation between repeat measures (*p* < 0.001). For these, repeat FGF-21 measures had CVs that ranged between 3.1% and 68.1% (median 18.0%), with the highest CV relating to duplicate measures that were 90 and 257 ng/L. However, in the case of a child with tyrosine hydroxylase deficiency in whom FGF-21 was >5700 ng/L, a repeat measurement a year later showed FGF-21 to be 70 ng/L. One other patient from whom five specimens were collected had serum FGF-21 concentrations that ranged from 227 to 421 ng/L (CV of 29.0%). In a further patient whose clinical conditions fluctuated, three FGF-21 measurements showed changes with results that reflected the clinical status of the patient (FGF-21 range of 800 to 1595 ng/L). 

**Figure 4:**
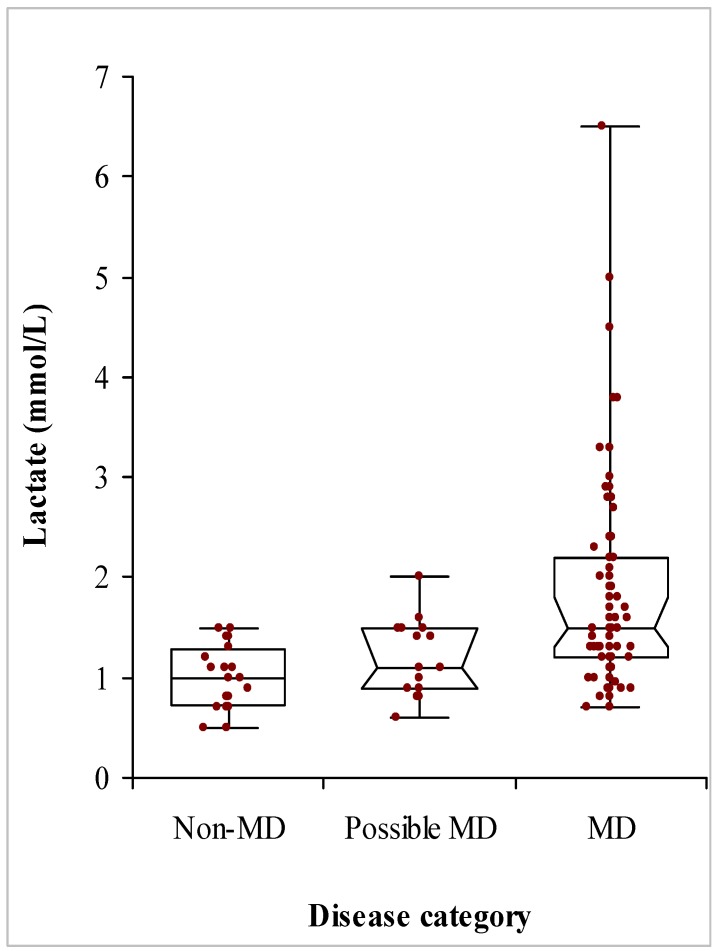
Distribution of plasma lactate concentrations in patients investigated for mitochondrial disease (MD). Patients were grouped according to the final diagnoses, with those who were likely to have MD by further investigations were still pending placed in the possible MD group.

**Figure 5:**
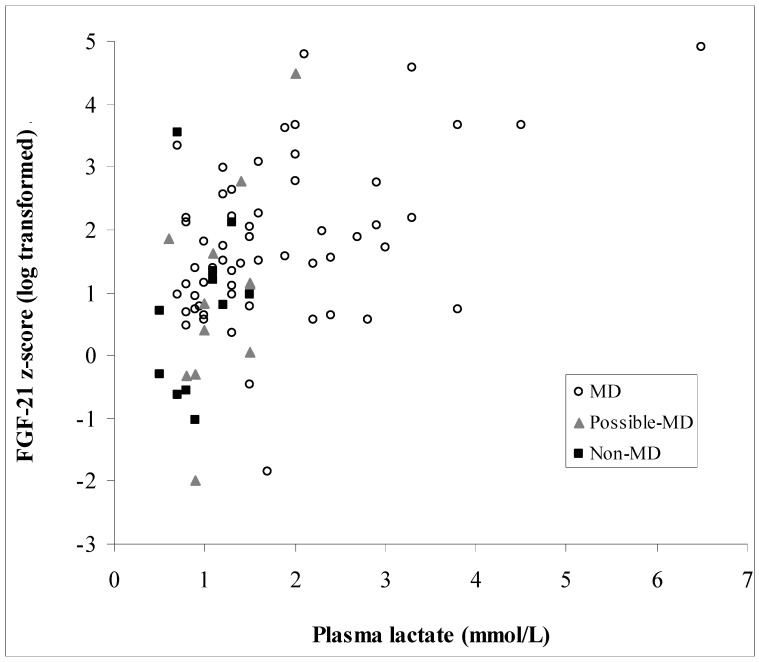
Relationship between log-transformed FGF-21 *z*-scores and plasma lactate in 80 patients (57 MD, 12 possible MD, and 11 non-MD), on whose specimens lactate values were available. A formula of FGF-21 *z*-score = 0.53*x* lactate + 0.84 describes the relationship between the two measures (*R*^2^ = 0.205).

There was no correlation between FGF-21 and plasma creatinine (*p* = 0.835). CK was measured in 66 patients, and there was no relationship between it and FGF-21 *z*-scores (*p* = 0.239). There was no difference between CK values in patients with and without (cardio) myopathy (*p* = 0.084) or patients with and without ophthalmoplegia (*p* = 0.275). Lactate was measured in 80 patients, 57 of whom had MD with median (IQR) lactate concentrations of 1.50 (1.23) mmol/L (range 0.7–6.5 mmol/L), compared with only 11 non-MD lactates that had a median (IQR) of 0.90 (0.48) mmol/L (range 0.5–1.5 mmol/L) (Figure 4). There was a significant positive association between FGF-21 *z*-scores and lactate (*p* < 0.0001; Figure 5). Patients without MD all had plasma lactate concentrations below 1.5 mmol/L, well within lactate reference interval, with a plasma lactate of 1.5 mmol/L displaying a sensitivity of 46% for MD. Figure 3 compares the ROC curves for FGF-21 *z*-score, lactate and the product of FGF-21 and lactate concentrations. There was no significant difference between the areas under the ROC curves, although both lactate and the arithmetic product of FGF-21 and lactate concentrations gave higher areas of 0.81 and 0.77, respectively. Amongst all patients investigated, the presence of (cardio) myopathy was associated with a higher plasma lactate (median (IQR) of 1.9 (1.5) mmol/L; *p* = 0.021), and so was ophthalmoplegia (median (IQR) of 1.5 (1.1) mmol/L; *p* = 0.047) compared with lactate in other patients (median (IQR) of 1.2 (0.6) mmol/L for both groups of patients without (cardio) myopathy and those without ophthalmoplegia).

## 4. Discussion

In search of better biochemical markers for MD, a few previous studies have suggested serum FGF-21 to have adequate sensitivity and specificity for it to be employed in MD diagnostic pathway, and for its values to correlate with disease severity [5,6,7,22]. In order to measure FGF-21 for assessing referred patients, we employed the same ELISA kit used by previous studies. We studied two sets of healthy populations’ FGF-21 data: one largely adult from the kit manufacturer, and a smaller one from local populations of adults and children. Differences between the results from the two sets of population made us use only the locally derived mean and SD values from normalised data, but this had limitations. Thus, although based on the external data, FGF-21 is affected by age, and the local population was too small to allow us to derive age-related values in adults. Nevertheless, based on separate log-normalised distributions, we used *z*-scores to assess the magnitude of increase in serum FGF-21 of patients.

We found serum FGF-21 concentrations to be higher in patients with MD compared with others. Those with mtDNA maintenance defects had the highest FGF-21, followed by patients with mtDNA rearrangements. Overall, however, there was a significant degree of overlap in FGF-21 values between MD and non-MD patients, such that FGF-21 *z*-score had an ROC curve area of 0.68. Patients with either mtDNA maintenance defects or mtDNA rearrangement had higher FGF-21. In this respect, our findings are consistent with those of the Finnish group [5], who found that serum FGF-21 discriminated most effectively from controls those patients with defects in mtDNA maintenance and single rearrangements. In particular, we did not find increased FGF-21 in the majority of our m.3243A>G patients, with only 6/29 (21%) having a *z*-score of over 2.0. This may reflect the contrast between our m.3243A>G patient population, which had mainly maternally inherited diabetes and deafness, and the previously published populations that were based on neurological disorders [23]. Nevertheless, although our data show that FGF-21 improves the biochemical investigation of patients suspected of having MD, they do not display the diagnostic powers that have been reported previously. Furthermore, FGF-21 was less useful in patients with non-myopathic disease affecting a single system, such as mitochondrial optic neuropathies. 

We also found that plasma lactate displayed an ROC curve that was at least as good as that of FGF-21 *z*-scores. Patients with myopathy also had higher lactate concentrations than others. However, the highest lactate concentration in the non-MD group was 1.5 mmol/L. Although this conferred a better diagnostic performance in our cohort, giving a specificity of 100% and a sensitivity of 46% for detecting MD, this was based on a small population of only 11 non-MD patients. Furthermore, unlike FGF-21 *z*-score of 2.8, which is well above the upper limit of normal values, a lactate of 1.5 mmol/L is well within the reference interval (up to 2.2 mmol/L), potentially posing problems in clinical practice with a true specificity that would be lower than seen here. Nevertheless, given the diagnostic use of plasma lactate, we assessed the power of the product of FGF-21 and lactate concentrations for detecting MD. However, this did not show any superiority over either analyte.

We measured FGF-21 routinely and in a non-discriminatory fashion in our patients and found several non-MD patients to have high FGF-21 values. FGF-21 is not specific to MD and has been shown to increase in many other conditions, including obesity, diabetes, fatty liver disease, and metabolic syndrome [24,25,26]. In contrast to mice [27], starvation and ketogenic states do not appear to increase FGF-21 significantly in humans [9], and we did not observe any association between serum FGF-21 and renal function, as has been described previously [28]. To what extent our data in non-MD patients has been influenced by such physiological factors is unknown. Furthermore, since FGF-21 increases significantly after fructose consumption [29], we propose that a more standardised approach to sample collection that records relevant clinical parameters and pre-analytical variables, such as feeding state, would be needed.

Other investigators have been able to demonstrate a positive correlation between FGF-21 levels and the Newcastle mitochondrial disease scale for adults (NMDAS) clinical assessment scale [30]. We do not routinely use NMDAS score and hence could not assess this. However, given that the majority of the individuals with both proven MD and high FGF-21 levels have ocular myopathies (100% and 87% of patients who were seen in the clinic with single rearrangements and defects of mtDNA maintenance, respectively), the test may be less sensitive than skilled clinical examinations.

Although FGF-21 has been described as a “mitokine”, its physiological role is uncertain. Under most physiological conditions the liver is the main contributor to circulating levels, followed by adipose tissue [31]. However, cardiac stresses may increase serum FGF-21 both in human cardiovascular disease [14] and in animal models of progressive MD [32,33]. The increase in circulating FGF-21 associated with human MD is believed to be a response to stress, originating from liver, adipose tissues, and skeletal muscle [34,35]. Although we did not measure respiratory chain activities in all patients, our data did not show any significant difference between FGF-21 values in patients with and without clinical evidence of significant myopathy. For example, we found an FGF-21 *z*-score of only 0.56 in a patient with complex-I deficiency due to mutations in the gene encoding an assembly factor, known as acyl-CoA dehydrogenase family member 9 (ACAD9). However, we found patients with ophthalmoplegia to have higher FGF-21 concentrations. Studies in mice suggest that mitochondrial dysfunction results in an increase in serum FGF-21 that is distinct from the PPARα-dependent response to starvation. Therefore, based on previous studies in mice, we suggest that FGF-21 increase may depend upon activating transcription factor 4 and the integrated stress response (ISR) [32,36], which itself is influenced by many factors including nutritional status and potentially the type of MD [22]. It is increasingly clear that several different mitochondrial stresses, including the mitochondrial unfolded protein response [37], mtDNA depletion, and inhibition of mitochondrial translation [38], can activate ISR. The type of mitochondrial stress caused by specific molecular defects that ultimately activate ISR-dependent FGF-21 expression may therefore underlie the variability of plasma FGF-21 levels observed in the MD group. Furthermore, its rather indirect relationship to MD limits its usefulness as a biomarker for the disease.

In general, we tended to find relatively consistent FGF-21 values in duplicate samples. Anecdotally, in two patients undergoing intensive care, the highest values were associated with respiratory deterioration (but without lactic acidosis), consistent with the high serum FGF-21 concentrations in patients undergoing intensive care [15]. Given the known relationship between FGF-21 and body weight [39], this type of response may play an important role both in progressive weight loss that is frequently observed in MD [40] and in the poor response of patients to critical care. Further investigation is needed in order to determine whether any apparent longitudinal change in serum FGF-21 may be related to the clinical course of the disease. If so, measuring FGF-21 may prove to be more useful for assessing disease progression and for the monitoring of the effects of therapies, rather than as a diagnostic biomarker. If having high FGF-21 levels benefit mitochondrial patients, the FGF-21 analogue that has been trialled as a drug treatment for hyperglycaemia and obesity [41,42] would also have potential as a therapy for MD.

In conclusion, our experience has shown that in clinical practice, FGF-21 measurements would be helpful in only around one-third of patients who are investigated in a specialist MD clinic. In our hands, a *z*-score of above 2.8 has given a specificity and a sensitivity of 93% and 21%, respectively. This indicates that although the test may not be used for the diagnosis of MD, its availability would affect the diagnostic pathway. In many patients, finding a high FGF-21 would suggest MD, and a targeted diagnostic approach in such patients would be preferable to next-generation sequencing. In the past, we used muscle biopsy to triage those patients needing detailed genetic investigation. Now, based on high serum FGF-21 values, we are able to identify those adult patients that would benefit from genetic investigations without prior muscle biopsy. The availability of this assay has thus modified our diagnostic pathway in adults, whereas muscle biopsy remains more important in children [43].

FGF-21 is now a useful adjunct to our clinical examination. Furthermore, because it is stable in samples at room temperature, we can analyse specimens sent in from peripheral clinics. However, further work is needed to determine whether it is useful in children, as currently there is insufficient reference data, and we saw a high proportion of false positives in this age group. Our clinic cohort was limited by the low number of disease controls having either non-mitochondrial myopathies or conditions that secondarily affect mitochondria. Further data are also needed to determine whether the apparent longitudinal changes in FGF-21 are related to the clinical course. Finally, growth and differentiation factor 15 (GDF-15) was recently identified in transcriptosome profiling and has been found to be a biomarker for MD. GDF-15 is held to have a superior sensitivity but a lower specificity compared with FGF-21 [22,44]. It remains to be seen whether combining FGF-21 with GDF-15 and conventional lactate measurements offers any advantage in stratifying patients investigated for MD.

## Figures and Tables

**Table 1:** Demographics and FGF-21 data in patients diagnosed with mitochondrial disease (MD), those without MD (non-MD), and patients with a possibility of having MD. FGF-21 *z*-scores are based on log-transformed values. Data on categories of MD, as well as those on patients with and without (cardio) myopathy and ophthalmoplegia (progressive external ophthalmoplegia or ptosis), have been given.

Diagnostic Category	*n*	Median Age (Range)	Median FGF-21 *z*-Score (95% C.I.)	FGF-21 *z*-Score IQR
Non-MD	27	45 (0.5–78)	0.86 (−0.39–1.80)	2.00
Possible MD	24	11 (0–63)	1.87 (1.81–2.78)	2.72
MD	104	39.5 (2–87)	1.72 (1.46–1.99)	1.65
-mtDMA maintenance defects	32	50 (7–87)	1.99 (1.46–2.73)	1.69
-mtDNA rearrangements	17	35 (2–82)	1.99 (1.56–2.76)	1.30
-mt DNA point mutations	40	40 (14–72)	1.40 (0.99–1.94)	1.26
-Partially characterised mitochondrial myopathy	8	22.5 (2–71)	1.64 (−0.47–4.15)	1.88
-Other autosomal MD	7	36 (3–56)	0.90 (0.56–3.56)	1.88
*Patients without (cardio)myopathy*	*117*	*37 (0–84)*	*1.13 (1.30–1.94)*	*1.85*
*Patients with (cardio)myopathy*	*39*	*40 (3–87)*	*1.74 (1.52–2.11)*	*1.35*
*Patients without ophthalmoplegia*	*93*	*35*	*1.33 (0.99–1.88)*	*1.88*
*Patients with ophthalmoplegia*	*63*	*43*	*1.74 (1.49–2.11)*	*1.51*

**Table 2:** Performance of log-transformed FGF-21 *z*-scores for diagnosing mitochondrial disease. FGF-21 *z*-scores refer to thresholds. Figures in brackets are 95% confidence intervals. PV is predictive value.

FGF-21 *z-*Score	Sensitivity	Specificity	Positive PV	Negative PV
1.35	0.65 (0.55–0.74)	0.70 (0.50–0.86)	0.90	0.35
2.82	0.20 (0.13–0.29)	0.93 (0.76–0.99)	0.91	0.23
